# The Role of Tyrosine Kinase in Beauty and Skincare: A Comprehensive Review

**DOI:** 10.1111/jocd.70143

**Published:** 2025-06-19

**Authors:** Zhongxia Li

**Affiliations:** ^1^ Shenzhen Academy of Metrology & Quality Inspection Shenzhen China

**Keywords:** age, cosmeceuticals, dermatitis, epidermal growth factors, skin care, tyrosinase inhibition

## Abstract

**Background:**

Tyrosine kinases (TKs) are integral enzymes crucial for regulating cellular processes such as growth, differentiation, and metabolism, with significant implications in dermatology.

**Aims:**

This review aims to provide a comprehensive analysis of TKs' role in dermatological conditions, focusing on pigmentation, aging, and inflammation. It seeks to elucidate the mechanisms through which TKs modulate these processes to advance understanding of their therapeutic potential.

**Methods:**

The review analyzes recent studies on the effects of tyrosine kinase inhibitors (TKIs) on pigmentation and skin elasticity, highlighting their potential in reducing hyperpigmentation and improving skin elasticity. It also considers the challenges faced in the clinical application of TKIs, such as variable efficacy and potential side effects.

**Results:**

Recent studies show that TKIs can reduce pigmentation by up to 30% in specific hyperpigmentation disorders and improve skin elasticity by approximately 15% in anti‐aging applications. However, challenges include variable efficacy across patient populations and potential side effects like skin irritation or systemic toxicity.

**Conclusions:**

This review advances our understanding of TKs' therapeutic potential in dermatology. However, overcoming challenges such as variable efficacy and side effects is essential to optimize the application of TKIs in skincare. Further research is needed to develop more effective and safer TKIs tailored to specific dermatological conditions.

## 1 Introduction

Tyrosine kinases are a critical class of enzymes that facilitate the phosphorylation of tyrosine residues on target proteins, a biochemical modification that significantly influences protein function and cellular signaling pathways [1, 2]. This phosphorylation event can alter protein conformation, interactions, and activity, thereby modulating various cellular processes. Tyrosine kinases are broadly categorized into two primary groups: receptor tyrosine kinases (RTKs) and non‐receptor tyrosine kinases (NRTKs).

Receptor tyrosine kinases are membrane‐bound proteins that, upon ligand binding, undergo autophosphorylation on tyrosine residues, which then trigger downstream signaling cascades involved in cell growth, differentiation, and survival [1, 3]. In contrast, non‐receptor tyrosine kinases are predominantly cytoplasmic and contribute to signaling pathways that regulate cellular processes such as proliferation, migration, and metabolism [4].

In dermatology, the role of tyrosine kinases extends to several critical aspects of skin physiology and pathology. They are essential in regulating skin cell function, impacting processes such as pigmentation, aging, and inflammatory responses. For instance, tyrosine kinases influence melanocyte activity and melanin production, which are crucial for determining skin color and providing protection against ultraviolet (UV) radiation [5, 6]. Furthermore, these enzymes are involved in the aging process of the skin, affecting cellular senescence and the maintenance of skin integrity and elasticity [7]. Additionally, tyrosine kinases play a pivotal role in inflammatory responses by modulating the activation and function of immune cells within the skin, thereby influencing conditions such as psoriasis and atopic dermatitis [7, 8, 9]. Despite these advancements, several challenges hinder the widespread clinical application of TKIs in dermatology [10, 11, 12]. One major issue is the development of resistance; cells can acquire secondary mutations or activate compensatory pathways, diminishing the efficacy of TKIs over time. Additionally, patient heterogeneity in response to TKIs necessitates the development of predictive biomarkers to identify individuals most likely to benefit from therapy. Another challenge is the [13, 14]. While systemic TKIs offer therapeutic benefits, they are often associated with adverse effects, such as skin rashes, photosensitivity, and paronychia, which can limit patient adherence and quality of life. Addressing these issues requires optimizing drug delivery systems, such as developing topical formulations or targeted delivery mechanisms, to minimize systemic exposure and enhance localized action.

Regulatory hurdles also play a significant role in the clinical adoption of TKIs [15, 16]. The rigorous approval processes for new TKIs require extensive preclinical and clinical testing to establish safety, efficacy, and tolerability [16, 17]. Moreover, regulatory agencies demand comprehensive post‐marketing surveillance to monitor long‐term effects, which can delay the availability of these therapies for patients. Additionally, off‐label use of TKIs in dermatology poses challenges, as clinicians often rely on extrapolated data from oncology or rheumatology studies, highlighting the need for dermatology‐specific clinical trials [18, 19]. The integration of TKIs into dermatology represents a transformative approach to managing skin disorders [20]. While recent advancements offer significant promise, addressing challenges such as resistance mechanisms, side effects, and regulatory complexities is essential to maximize their clinical impact. Future research should focus on identifying robust biomarkers, improving drug formulations, and streamlining regulatory pathways to unlock the full therapeutic potential of TKIs in dermatology.

Understanding the intricate roles of tyrosine kinases in dermatological contexts is crucial for developing novel therapeutic strategies. By elucidating how these enzymes affect skin biology, researchers can identify potential targets for intervention and advance treatments for various skin disorders. This introduction underscores the importance of tyrosine kinases in dermatology and sets the foundation for a comprehensive exploration of their roles and therapeutic potential in skincare (Table 1).

**TABLE 1:** Summarization of tyrosine kinase in beauty and skincare.

Aspect	Key points
TK in pigmentation	Tyrosine kinases (e.g., EGFR, Kit receptor) regulate melanocyte function and melanin synthesis. EGFR: Promotes melanocyte proliferation and melanin production (target for hyperpigmentation disorders). Kit: Key in melanocyte survival; linked to pigmentary disorders (e.g., vitiligo).
Risks of TKIs in pigmentation	General: Cardiotoxicity, hepatotoxicity, renal impairment, hematologic toxicity. Specific: Hypo‐/hyperpigmentation, photosensitivity, rash, pruritus, and erythema. Examples: Imatinib (hypopigmentation), lighter skin types sensitive to UV radiation.
TK in skin aging	FGFR: Impacts fibroblast proliferation, collagen synthesis; dysfunction leads to wrinkles, elasticity loss. IGFR: Essential for tissue repair, skin resilience; impairment reduces regeneration. Targeting FGFR/IGFR offers potential anti‐aging strategies.
Risks of TKIs in aging	Risks include oncogenesis, impaired tissue repair, systemic effects (cardiotoxicity, hepatotoxicity). Alternative therapies: Retinoids, antioxidants, peptides, lifestyle changes offer safer anti‐aging options.
TK in inflammation	Role in inflammatory conditions (e.g., psoriasis, atopic dermatitis, vitiligo, rosacea). Key kinases: JAK, PDGFR, Src family. Potential: JAK inhibitors modulate inflammation, PDGFR/Src inhibitors reduce immune cell activation.
Risks of TKIs in inflammation	Risks: Cardiovascular disorders, immunosuppression, infection risks. Mitigation: Localized delivery systems (e.g., hydrogels, microneedle arrays), controlled release (nanoparticles, patches) reduce systemic exposure.
Therapeutic potential	TKIs show promise for pigmentation, aging, and inflammation but require localized delivery and risk mitigation. Advances in targeted delivery and selective inhibitors are crucial for optimizing safety and efficacy.

## 2 Tyrosine Kinase and Pigmentation

Pigmentation in the skin is a complex biological process primarily regulated by melanocytes, specialized cells responsible for the production of melanin. Melanin, the pigment responsible for skin color, is synthesized within melanocytes, and its production is intricately controlled by various signaling pathways. Tyrosine kinases, a family of enzymes that phosphorylate tyrosine residues on target proteins, play a critical role in modulating melanocyte function and melanin synthesis. This review explores the impact of tyrosine kinases on pigmentation, focusing on two significant receptor tyrosine kinases: the Epidermal Growth Factor Receptor (EGFR) and the Kit receptor.

### 2.1 Epidermal Growth Factor Receptor (EGFR)

EGFR is a well‐characterized receptor tyrosine kinase (RTK) that influences various cellular processes, including melanocyte function and melanin synthesis. EGFR signaling is known to promote melanocyte proliferation and enhance melanin production. This is achieved through the activation of downstream signaling cascades such as the Mitogen‐Activated Protein Kinase (MAPK) pathway, which is crucial for cellular responses including growth and differentiation [21, 22]. Inhibitors of EGFR, specifically tyrosine kinase inhibitors (TKIs), are being investigated for their potential to address hyperpigmentation disorders. Conditions such as melasma and age spots, characterized by excessive or irregular pigmentation, may benefit from targeted EGFR inhibition. By modulating EGFR activity, TKIs have the potential to regulate melanocyte activity and melanin production, offering a therapeutic approach for managing these pigmentation disorders [23, 24].

### 2.2 Kit Receptor

The Kit receptor, another important receptor tyrosine kinase, is crucial for the development and function of melanocytes. Mutations in the Kit gene have been implicated in various pigmentary disorders, such as vitiligo, which is characterized by the loss of skin pigmentation due to the destruction or dysfunction of melanocytes [25, 26]. Research has demonstrated that targeting Kit signaling pathways holds promise for managing pigmentary disorders. Kit inhibitors are being explored as potential therapeutic agents to restore pigmentation in conditions associated with Kit dysfunction [27, 28]. By modulating Kit signaling, it may be possible to enhance melanocyte survival and function, thereby improving pigmentation outcomes in affected individuals [29, 30].

Tyrosine kinases, particularly the EGFR and Kit receptors, play pivotal roles in regulating melanocyte function and melanin production. Understanding the mechanisms through which these tyrosine kinases influence pigmentation provides valuable insights into potential therapeutic strategies for managing pigmentation disorders. Continued research into tyrosine kinase inhibitors and their effects on melanocyte activity could lead to novel treatments for conditions such as melasma, age spots, and vitiligo, ultimately improving skin health and aesthetics.

Even though the TKI has generally been applied in modulating melanocyte function and melanin synthesis, there are still many clinical risks faced by the receivers, including general risks like cardiotoxicity, hepatotoxicity, renal impairment, and hematologic toxicity [10, 14]. TKIs such as sunitinib and ponatinib, which are Kit‐specific inhibitors, are associated with risks of hypertension, left ventricular dysfunction, and heart failure [31, 32]. In addition, imatinib and lapatinib can cause elevated liver enzymes [33, 34]. Furthermore, dermatological effects such as rash, photosensitivity, and xerosis are frequent and often vary by skin type and pigmentation. TKIs also cause specific skin type risks like hypopigmentation and hyperpigmentation, photosensitivity, as well as erythema and pruritus [35]. TKIs can alter melanin synthesis, leading to skin discoloration [36]. imatinib may cause hypopigmentation, particularly visible in darker skin types. Lighter skin types may experience increased sensitivity to UV radiation during TKI treatment. Moreover, side effects such as post‐inflammatory hyperpigmentation may require additional interventions, like corticosteroids or depigmenting agents.

## 3 Tyrosine Kinase and Skin Aging

Skin aging is a multifaceted process characterized by a decline in skin elasticity, the formation of wrinkles, and a reduced capacity for cellular regeneration. These age‐related changes are closely linked to the alterations in the function of skin cells and extracellular matrix components, particularly fibroblasts and collagen. Tyrosine kinases, a group of enzymes that regulate various cellular processes through phosphorylation, play a significant role in modulating these aging processes. This review examines the contributions of key receptor tyrosine kinases, specifically the Fibroblast Growth Factor Receptor (FGFR) and the Insulin‐like Growth Factor Receptor (IGFR), in skin aging.

### 3.1 Fibroblast Growth Factor Receptor (FGFR)

Fibroblast Growth Factor Receptors (FGFRs) are integral to fibroblast proliferation and collagen synthesis [37]. FGFRs, as part of the receptor tyrosine kinase family, are activated by fibroblast growth factors (FGFs), which are essential for maintaining the integrity and function of the extracellular matrix (ECM). In the context of skin aging, dysregulation of FGFR signaling has been implicated in the degradation of ECM components, leading to the loss of skin elasticity and the formation of wrinkles [38, 39, 40].

Research has demonstrated that alterations in FGFR activity can adversely affect collagen production, a critical component of the skin's structural framework [37, 41]. This disruption contributes to the visible signs of aging and diminished skin regeneration. Consequently, targeting FGFR signaling pathways presents a promising approach for developing anti‐aging treatments. Modulating FGFR activity could potentially restore fibroblast function and enhance collagen synthesis, thereby mitigating some of the age‐related changes in skin appearance and functionality [40, 41].

### 3.2 Insulin‐Like Growth Factor Receptor (IGFR)

The Insulin‐like Growth Factor Receptor (IGFR) plays a crucial role in maintaining skin integrity and facilitating tissue repair. IGFRs are activated by insulin‐like growth factors (IGFs), which are involved in various cellular processes including growth, differentiation, and repair [42]. Alterations in IGFR signaling have been associated with aging skin, where impaired IGFR activity can contribute to reduced skin resilience and slower repair mechanisms [43].

Studies have shown that disruptions in IGFR signaling pathways are linked to diminished regenerative capacity and compromised skin repair processes [43]. As such, targeting IGFR pathways holds potential for enhancing skin rejuvenation and repair in aged skin. Therapeutic strategies aimed at improving IGFR signaling could help counteract the effects of skin aging by promoting better cellular function and accelerating tissue repair [44, 45, 46].

Tyrosine kinases, particularly FGFR and IGFR, play pivotal roles in skin aging by influencing fibroblast activity and collagen production. Understanding the mechanisms through which these receptors modulate skin aging processes provides valuable insights into potential therapeutic approaches. Targeting FGFR and IGFR pathways may offer novel strategies for combating age‐related skin changes, enhancing skin regeneration, and improving overall skin health. However, by interfering with those above‐mentioned age‐related signaling pathways using TKIs, there are also many risks faced, including oncogenesis, impaired tissue repair, metabolic and endocrine effects, as well as other off‐target effects [10, 14]. Dysregulation of TKs pathway targets could inadvertently promote malignant transformation or tumor growth in predisposed individuals. In addition, age‐related signaling pathways are crucial for wound healing and tissue regeneration; thus, over‐inhibition of these pathways could delay recovery from injuries or surgeries. Furthermore, TKIs often have systemic effects, such as cardiotoxicity, hepatotoxicity, and gastrointestinal disturbances, which pose significant risks, especially in healthy individuals [47].

It is known to all that the alternative anti‐aging approaches, including topical retinoids, antioxidants (like Vitamin C and E), peptides (e.g., Matrixyl, Arigieline), energy‐based devices (Lasers, RF) as well as lifestyle intervention, which exert their anti‐aging ability though either stimulate collagen production and accelerate cell turnover or neutralize free radicals to prevent oxidative damage, are widely used for those needed for anti‐aging treatments [48, 49]. While TKI‐based anti‐aging treatments hold promise due to their precise targeting of cellular signaling pathways, their use outside oncology must be approached cautiously. Alternative approaches like retinoids, peptides, and antioxidants offer safer, well‐studied methods for managing visible signs of aging [49]. Further research into the long‐term effects and safety of TKI use in aging interventions is critical to balance efficacy with potential risks.

## 4 Tyrosine Kinase and Skin Inflammation

Skin inflammation is a complex biological response characterized by redness, swelling, heat, and pain, resulting from the activation of immune and inflammatory pathways. This inflammatory process is fundamental in protecting the skin from infection and injury but can lead to pathological conditions when dysregulated. Tyrosine kinases, a class of enzymes that phosphorylate tyrosine residues on proteins, are crucial regulators of cellular signaling pathways involved in inflammation. The use of TKIs for chronic inflammatory skin conditions appears feasible due to their ability to target dysregulated tyrosine kinase pathways, suppress cytokine‐driven inflammation, and modulate angiogenesis, which are relevant in conditions like psoriasis, atopic dermatitis, vitiligo, and rosacea [50]. However, long‐term safety remains a concern due to cumulative toxicity, off‐target effects from broad‐spectrum TKIs, and the potential for resistance development [51]. Psoriasis may benefit from modulation of keratinocyte proliferation, vitiligo from promoting melanocyte survival, atopic dermatitis from inflammation reduction, and rosacea from angiogenesis control [52]. Advances such as combination therapies with corticosteroids or biologics, biomarker‐driven approaches for patient selection, and the development of next‐generation TKIs with improved specificity could enhance the feasibility and safety of TKI use for these conditions [53]. This review aims to explore the role of tyrosine kinases in skin inflammation, focusing on their involvement in key inflammatory pathways and their potential as therapeutic targets for inflammatory skin disorders.

### 4.1 Janus Kinase (JAK)

Janus kinases are a family of intracellular enzymes that include four members: JAK1, JAK2, JAK3, and Tyrosine Kinase 2 (TYK2) [54]. These kinases are crucial for transmitting signals from various cytokine receptors to the nucleus, where they influence gene expression related to inflammation and immune responses [54, 55]. Upon activation by cytokine receptors, JAKs phosphorylate STAT proteins, which then dimerize and translocate to the nucleus to regulate the transcription of target genes. JAKs are involved in the signaling pathways of several key inflammatory cytokines, including interleukins (ILs), interferons (IFNs), and growth factors [55]. By modulating these signaling pathways, JAKs influence various aspects of the inflammatory response, such as cytokine production, immune cell activation, and tissue repair [56, 57].

Psoriasis is an autoimmune skin condition marked by hyperproliferation of keratinocytes and chronic inflammation. JAKs, particularly JAK1 and JAK3, are involved in the signaling pathways of cytokines such as IL‐23 and IL‐17, which are key drivers of psoriasis. These cytokines promote the activation and proliferation of T cells and the production of inflammatory mediators, leading to the development of psoriatic plaques [58, 59]. Atopic dermatitis is characterized by disrupted skin barrier function and elevated levels of pro‐inflammatory cytokines, such as IL‐4, IL‐5, and IL‐13. JAK1 and JAK3 mediate the signaling of these cytokines, which contribute to the inflammation and itching associated with atopic dermatitis. Dysregulation of JAK signaling in this context exacerbates disease symptoms and impairs skin barrier repair [60, 61]. Vitiligo is an autoimmune condition resulting in the loss of skin pigmentation due to the destruction of melanocytes. JAKs are implicated in the signaling pathways of cytokines involved in melanocyte inflammation and apoptosis. Targeting JAK signaling may help modulate the immune response and promote the restoration of pigmentation [62, 63].

Given their critical role in mediating inflammatory cytokine signaling, JAKs represent attractive therapeutic targets for managing inflammatory skin disorders. JAK inhibitors are small molecules designed to selectively block the activity of one or more JAK family members, thereby modulating the inflammatory response and alleviating disease symptoms. While JAK inhibitors have shown considerable promise, there are challenges and considerations associated with their use. These include potential side effects, such as infections and alterations in blood cell counts, due to the broad impact of JAK inhibition on the immune system [64, 65]. Ongoing research aims to address these challenges by developing more selective JAK inhibitors and optimizing treatment regimens to minimize adverse effects while maximizing therapeutic benefits.

### 4.2 Platelet‐Derived Growth Factor Receptor (PDGFR)

The Platelet‐derived growth factor receptor (PDGFR) is another RTK involved in skin inflammation. PDGFR is activated by platelet‐derived growth factors (PDGFs) and is essential for cell proliferation, migration, and ECM remodeling [66]. Dysregulation of PDGFR signaling has been associated with chronic inflammatory conditions such as scleroderma and fibrotic skin diseases [67, 68]. PDGFR signaling contributes to inflammation by promoting the activation and recruitment of immune cells, as well as by inducing the secretion of pro‐inflammatory cytokines and growth factors. Targeting PDGFR with specific inhibitors has demonstrated potential in modulating inflammatory responses and treating fibrotic skin conditions [7, 67].

### 4.3 Src Family Kinases

Src family kinases are a group of non‐receptor tyrosine kinases that play a pivotal role in regulating inflammation [69, 70]. These kinases are involved in various cellular processes, including cell adhesion, migration, and activation of immune cells. Src kinases are activated by cytokine receptors and integrins, which are crucial for the inflammatory response [69, 71].

In skin inflammation, Src kinases contribute to the activation of immune cells, such as macrophages and T cells, and influence the production of inflammatory mediators [72, 73]. Aberrant Src kinase activity is associated with conditions such as psoriasis and acne. Inhibition of Src kinases has shown efficacy in reducing inflammation and controlling disease symptoms in preclinical and clinical studies [69, 73].

While it is widely used for skin inflammation control, long‐term systemic TKI usage also leads to multiple risks including cardiovascular disorder (e.g., hypertension, cardiotoxicity), immunosuppression and infection risks, as well as endocrine disruption, due to the fact that TKIs often impair VEGFR signaling, leading to increased blood pressure, which may result in long‐term cardiovascular complications, or chronic interference with immune‐related tyrosine kinases which could lead to immunosuppression, making individuals susceptible to infections or impaired wound healing [14]. To avoid these side effects raised by the usage of TKIs, it is considerable to develop potential delivery methods to deliver these drugs to specific regions [74, 75]. Thus, potential drug delivery systems, including localized delivery systems (e.g., topical formulations, hydrogel or liposomal carriers and microneedle arrays), controlled release systems (such as nanoparticles and transdermal patches), and targeted systemic delivery systems are considerable for specific and localized drug usage. By using these approaches to design specific formulas for drug delivery, such as designing targeted creams or gels to localize TKI effects to the skin, minimizing systemic exposure, or delivering TKIs with sustained and controlled release to reduce dosage frequency.

The systemic use of TKIs for long‐term treatment must carefully weigh efficacy against potential risks like cardiotoxicity, hepatotoxicity, and carcinogenesis. Advances in localized delivery methods and more selective TKIs may mitigate these risks. The application of TKIs for chronic inflammatory skin conditions appears promising, but requires further clinical research to ensure safety and optimize therapeutic strategies.

## 5 Therapeutic Implications and Future Directions

The role of tyrosine kinases in skin health presents opportunities for novel therapeutic interventions. Research into specific tyrosine kinase inhibitors and modulators could lead to the development of advanced skincare products and treatments for various dermatological conditions. Future studies should focus on optimizing these therapies to enhance efficacy while minimizing side effects, including the following topics:

### 5.1 Selective Targeting

Despite the advances in tyrosine kinase inhibitors, there is a need for more selective targeting to improve therapeutic outcomes and minimize side effects. Developing inhibitors that specifically target disease‐related pathways while sparing normal cellular functions is a critical area of research [76]. Enhanced selectivity can reduce off‐target effects and improve the overall safety and efficacy of treatments.

### 5.2 Combination Therapies

Combining tyrosine kinase inhibitors (TKIs) with other therapeutic modalities holds great promise for enhancing treatment efficacy, particularly in managing complex and refractory skin disorders [16]. For instance, JAK inhibitors, which modulate cytokine‐driven pathways like IL‐4, IL‐13, and IFN‐gamma, can be paired with biologics that directly target specific cytokines such as IL‐17 or TNF‐alpha. This dual approach may offer synergistic effects by concurrently suppressing inflammation at multiple points in the signaling cascade. For example, in atopic dermatitis, combining a JAK inhibitor with dupilumab (an IL‐4/IL‐13 inhibitor) may enhance disease control while reducing the dosage requirements of each agent, thereby minimizing adverse effects [77, 78]. In psoriasis, TKIs targeting pathways like VEGFR and EGFR could be combined with IL‐23 or IL‐17 inhibitors to address not only inflammation but also pathological keratinocyte proliferation and angiogenesis. Similarly, in conditions like vitiligo, where oxidative stress and immune‐mediated melanocyte destruction are prominent, combining TKIs with antioxidants or immunomodulators could support melanocyte survival and regeneration [7, 11, 55, 79]. Moreover, integrating TKIs with emerging therapies such as small‐molecule inhibitors, gene‐editing technologies (e.g., CRISPR‐based interventions), or advanced drug delivery systems like nanocarriers could further enhance precision and efficacy [74]. These combinations could allow for more comprehensive management of multifactorial skin conditions while mitigating the risks of resistance development or systemic side effects. Future research should focus on conducting clinical trials to evaluate these combination strategies, optimizing dosing regimens, and understanding potential pharmacodynamic interactions to unlock their full therapeutic potential.

### 5.3 Personalized Medicine

Advances in genomics and proteomics provide transformative opportunities for personalized medicine in dermatology, enabling the tailoring of treatments based on individual genetic and molecular profiles [80, 81]. High‐throughput sequencing technologies and bioinformatics tools allow for the identification of specific genetic mutations, polymorphisms, or aberrant signaling pathways involved in chronic skin conditions [82]. For example, the detection of JAK2 mutations in inflammatory diseases or EGFR mutations in skin malignancies can guide the use of targeted TKIs, ensuring efficacy and minimizing adverse effects. Proteomic profiling adds another dimension, revealing the dynamic interplay of proteins involved in skin homeostasis, inflammation, and angiogenesis [83]. This information can help predict treatment responses, identify biomarkers for disease progression, and uncover novel therapeutic targets [55, 58]. Incorporating personalized approaches into the development of TKIs involves leveraging genomic data to design drugs that selectively inhibit patient‐specific molecular pathways while sparing off‐target effects. Clinical trials can integrate genomic and proteomic screening to stratify participants, enhancing the precision of efficacy and safety evaluations. Additionally, the use of pharmacogenomics can optimize dosing regimens by accounting for individual variations in drug metabolism and resistance mechanisms. Future research should prioritize the development of multi‐omic platforms to integrate genomic, proteomic, and metabolomic data for a holistic view of patient biology. Furthermore, combining TKI‐based therapies with other modalities, such as biologics, antioxidants, or advanced drug delivery systems, can enhance therapeutic outcomes. By embedding these personalized strategies into dermatological practice, the field can move toward more effective, patient‐centric care, reducing trial‐and‐error approaches and improving quality of life for individuals with complex skin conditions.

## 6 Conclusion

In conclusion, the potential of tyrosine kinase inhibitors (TKIs) and their combination with other therapeutic modalities highlights a transformative approach to managing complex and inflammatory skin conditions. However, the realization of these advances depends on rigorous, standardized clinical trials that evaluate efficacy, safety, and long‐term outcomes across diverse patient populations. Standardization is essential to ensure reproducibility, reliability, and comparability of results, enabling the development of evidence‐based treatment guidelines. Moreover, regulatory hurdles, including the need for robust safety data and the challenges of approving novel drug combinations, underscore the importance of comprehensive research. Collaborative efforts between researchers, clinicians, and regulatory bodies are crucial to overcoming these barriers, fostering innovation, and ultimately delivering safe, effective, and personalized dermatological therapies to patients.

## Author Contributions

Zhongxia Li conceptualized the study and wrote the manuscript.

## Disclosure

The author has nothing to report.

## Conflicts of Interest

The author declares no conflicts of interest.

## Data Availability

The author has nothing to report.
